# Risk factors for delayed wound healing after anal fistula surgery: Protocol of a meta-analytic study

**DOI:** 10.1371/journal.pone.0329030

**Published:** 2025-07-28

**Authors:** Zubing Mei, Peixin Du, Ye Han, Yin Qu, Huili Tang, Yan Li, Huiyan Gao, Qingming Wang, De Zheng

**Affiliations:** 1 Department of Anorectal Surgery, Shuguang Hospital, Shanghai University of Traditional Chinese Medicine, Shanghai, China; 2 Anorectal Disease Institute of Shuguang Hospital, Shanghai, China; 3 Department of Anorectal Surgery, Gongli Hospital of Shanghai Pudong New Area, Shanghai, China; 4 Beicai Community Health Service Center, Pudong New District, Shanghai, China; Government Gousia Hospital, DHS, Srinagar, INDIA

## Abstract

**Introduction:**

Delayed wound healing (DWH) following anal fistula surgery is a common complication that prolongs recovery, increases patient morbidity, and imposes significant healthcare costs. Potential risk factors such as diabetes, smoking, fistula complexity, and surgical techniques have been suggested in individual studies, yet no comprehensive synthesis exists to guide clinical practice. This study aims to identify and evaluate risk factors associated with DWH after anal fistula surgery by combining existing evidence and grading the evidence.

**Methods and analysis:**

This study will follow the Preferred Reporting Items for Systematic Review and Meta-Analysis Protocols (PRISMA-P) guidelines. We will search PubMed, Embase, Cochrane Library, Web of Science, and grey literature databases from inception to March 2025, with no language restrictions. Observational studies (cohort and case-control) reporting risk factors for DWH, defined as incomplete healing beyond 6–12 weeks post-surgery, will be included. Two independent reviewers will screen titles/abstracts, perform full-text reviews, extract data regarding study design, sample size, risk factors and outcomes, and assess risk of bias using the Newcastle-Ottawa Scale (NOS). Primary outcomes will include odds ratios (OR) or relative risks (RR) for factors such as comorbidities, lifestyle factors, and operative approaches. A random-effects meta-analysis will pool effect estimates if heterogeneity (I² < 50%) permits; otherwise, a narrative synthesis will be conducted. Subgroup analyses will explore differences by study design and patient characteristics, with publication bias assessed using funnel plots and Egger’s test. The certainty of evidence will be evaluated with the Grading of Recommendations Assessment, Development and Evaluation (GRADE) approach.

**Discussion:**

This study’s strengths include its comprehensive search strategy and rigorous methodology, providing a robust synthesis of risk factors. Clinically, identifying modifiable risk factors could enhance preoperative optimization and postoperative care, reducing delayed healing rates. Future studies should standardize definitions of delayed healing and explore under-investigated factors like wound care techniques or microbiome influences to refine risk prediction models.

**Registration:**

PROSPERO CRD420251013602

## Introduction

Anal fistula, a chronic abnormal communication between the anal canal and perianal skin, is a prevalent condition with an incidence of approximately 8.6–10 per 100,000 individuals annually, predominantly affecting males [1,2]. Surgical intervention remains the cornerstone of treatment, with techniques such as fistulotomy, seton placement, and advancement flaps aimed at eradicating the fistula tract while preserving continence [3–8]. However, delayed wound healing following these procedures is a frequent complication, reported in 10–30% of cases, leading to prolonged recovery, recurrent infections, and reduced quality of life [9–13]. This issue not only burdens patients but also imposes significant economic costs on healthcare systems, with extended outpatient follow-ups and additional interventions often required [14,15].

Delayed wound healing after anal fistula surgery is typically defined as incomplete epithelialization or persistent wound discharge beyond 6–12 weeks post-operatively, though definitions vary across studies [16–18]. The healing process is complex, influenced by a interplay of local tissue factors, systemic conditions, and surgical variables. Recent studies have identified several potential risk factors contributing to this complication. Patient-related factors, such as diabetes mellitus and smoking, have been consistently associated with impaired wound repair in various surgical contexts, including anal fistula surgery [9,18,19]. A cohort study by Tang et al. reported that diabetic patients had a 2.5-fold increased odds of delayed healing following fistulotomy (OR 2.5, 95% CI 1.8–3.4) [19]. Similarly, smoking has been linked to reduced tissue oxygenation and delayed collagen synthesis [20], with several studies confirming its adverse impact on wound outcomes [21,22].

Fistula-specific characteristics also play a criticalc role. Complex fistulas, including those with multiple tracts or supralevator extensions, are associated with higher rates of delayed healing due to increased tissue disruption and infection risk [18,23]. Kang et al. found that patients with high transsphincteric fistulas expcerienced higher rate of healing delays than that of simple fistulas [24]. Surgical technique further influences outcomes, with fistulotomy generally yielding faster healing than flap procedures, though at the cost of higher incontinence rates [25,26]. Additionally, postoperative infection, often exacerbated by poor wound care or microbial resistance, has been implicated as a key contributor, with rates as high as 25% in some series [27,28].

Systemic inflammatory conditions, such as inflammatory bowel disease (IBD), particularly Crohn’s disease, further complicate healing in anal fistula patients. Although distinct from idiopathic fistulas, Crohn’s-related fistulas share overlapping risk profiles, with studies showing that active inflammation and immunosuppressive therapy (e.g., anti-TNF agents) can delay healing by up to 50% [29]. However, even in non-IBD populations, elevated inflammatory markers like C-reactive protein (CRP) have been correlated with poorer outcomes [30,31]. Nutritional status is another emerging factor [32], with a study by Ge et al. reporting that hypoalbuminemia (<30 g/L) doubled the risk of major complications including delayed healing [33].

Despite the growing body of literature, evidence on crisk factors for DWH remains fragmented. Many studies are small, single-center investigations with heterogeneous definitions of delayed healing and inconsistent adjustment for confounders. For example, a systematic review by An et al. focused solely on surgical techniques and postoperative complications, omitting patient-level risk factors like comorbidities [25]. Similarly, another meta-analysis by Mei et al. addressed recurrence rates but not healing delays [9]. This lack of a comprehensive synthesis hinders the development of evidence-based strategies to mitigate this complication.

The socioeconomic implications of DWH are substantial. In Sweden, total discounted costs for anal fistula were €5,561 per patient, with delayed healing doubling these expenses due to additional treatments and lost productivity [34]. In low-resource settings, prolonged recovery exacerbates disparities in care access, underscoring the need for targeted interventions [35]. Identifying modifiable risk factors could inform preoperative optimization (e.g., smoking cessation programs) and postoperative management (e.g., enhanced wound care protocols), potentially reducing morbidity and costs.

Given the clinical and economic burden, coupled with the absence of a systematic evaluation, there is an urgent need to consolidate evidence on risk factors for DWH after anal fistula surgery. This study aims to address this gap by synthesizing data from observational studies to identify both patient-related and procedure-related predictors. By providing a robust evidence base, this study seeks to guide risk stratification, enhance surgical decision-making, and inform future research into preventive strategies, ultimately improving patient outcomes in this challenging condition.

## Methods

### Registration

The review is schedulecd to commence on 18 March 2025, and conclude by 31 January 2026. The report of this systematic review protocol adheres to the Preferred Reporting Items for Systematic Review and Meta-Analysis Protocols (PRISMA-P) guidelines [36] (S1 Table) and draws on the second edition of the Cochrane Handbook for Systematic Reviews of Interventions [37]. The protocol has been prospectively registered on PROSPERO (registration number. CRD420251013602). The final reporting of the systematic review and meta-analysis will follow the PRISMA 2020 statement [38].

### Eligibility criteria

Eligibility criteria are defined using an adapted PICOS framework (P – population, I – interventions/risk factors, C – comparator, O – outcomes, S – study design).

### Population

The population includes adults (≥18 years) undergoing surgery for anal fistula, encompassing only cryptoglandular anal fistulas. Studies focusing exclusively on Crohn’s disease-related fistulas will be excluded to maintain focus on idiopathic anal fistulas, though mixed populations will be considered if subgroup data are available.

### Risk factors

Risk factors will include patient-related factors (e.g., diabetes, smoking, obesity, nutritional status) and procedure-related factors (e.g., surgical technique, postoperative infection), as commonly reported in the literature [9,11,18]. These will be assessed as predictors of DWH, defined as incomplete epithelialization or persistent wound discharge beyond 6–12 weeks post-surgery. Potential risk factors and their typical measurement methods are detailed in Table 1.

**Table 1:** Potential Risk Factors for Delayed Wound Healing after Anal Fistula Surgery.

Risk Factor	Category	Definition/Description	Measurement Method	Source/Reference
Diabetes Mellitus	Patient-related	Chronic hyperglycemia impairing wound healing via microvascular damage and reduced angiogenesis	HbA1c (%), fasting glucose (mmol/L)	[19,44]
Smoking	Patient-related	Nicotine-induced vasoconstriction reducing tissue oxygenation and fibroblast function	Pack-years, current smoker (yes/no)	[20–22]
Obesity	Patient-related	Chronic inflammation and adipokine dysregulation increasing complication risk	BMI (kg/m²), waist circumference (cm)	[4,46,47]
Nutritional Status	Patient-related	Hypoalbuminemia limiting protein availability for tissue repair	Serum albumin (g/L), prealbumin (mg/dL)	[33]
Fistula Complexity	Procedure-related	High transsphincteric, supralevator, or branching tracts increasing tissue trauma	MRI/TRUS classification, number of tracts	[16,24]
Surgical Technique	Procedure-related	Variations (e.g., fistulotomy vs. flap) affecting healing speed and continence	Procedure type (categorical)	[25,26]
Postoperative Infection	Procedure-related	Microbial persistence triggering inflammation and delaying granulation	Wound culture, clinical signs (yes/no)	[27,28]
Inflammatory Markers	Patient-related	Elevated systemic inflammation impairing healing (non-IBD context)	CRP (mg/L), ESR (mm/h)	[30,31]

**Abbreviations**: BMI, body mass index; CRP, C-reactive protein; ESR, erythrocyte sedimentation rate; HbA1c, glycated hemoglobin; MRI, magnetic resonance imaging; TRUS, transrectal ultrasonography. References correspond to the manuscript’s citation list.

### Comparator

The comparison will involve the presence versus absence of each risk factor within studies reporting multiple predictors, facilitating the evaluation of their independent and relative contributions to DWH where data permit.

### Outcomes

The primary outcome is the incidence of DWH, measured as a dichotomous event (yes/no) at a minimum follow-up of 6–12 weeks post-surgery. Secondary outcomes include time to complete healing (in weeks) and wound-related complications (e.g., infection, dehiscence), as reported in primary studies using validated measures where available. Outcome definitions and measurement tools are outlined in Table 2.

**Table 2:** Outcome Measures for Delayed Wound Healing.

Outcome	Type	Definition	Measurement Tool	Time Point
Incidence of DWH	Primary	Incomplete epithelialization or persistent discharge beyond 6–12 weeks	Dichotomous (yes/no), clinical assessment	≥6–12 weeks post-op
Time to Complete Healing	Secondary	Duration from surgery to full epithelialization without discharge	Continuous (weeks), patient records	Variable (study-specific)
Wound-Related Complications	Secondary	Infection, dehiscence, or other healing disruptions	Categorical (type), clinical diagnosis	≥6–12 weeks post-op

**Abbreviations**: DWH, delayed wound healing; post-op, postoperative. Outcomes align with definitions from Garg et al. [16] and Ommer et al. [17].

### Study design

Observational cohort and case-control studies will be included, capturing both single-factor and multifactor associations with DWH.

### Exclusion criteria

Randomized controlled trials will be excluded due to their focus on intervention efficacy rather than naturalistic risk factor assessment. Case reports, case series, and studies lacking clear definitions of delayed healing will be excluded. Studies on pediatric populations (<18 years), Crohn’s disease-specific fistulas without idiopathic fistula data, or non-surgical treatments will also be excluded.

### Search strategy

Databases including PubMed, Embase (via Embase.com), Cochrane Library, Web of Science, and grey literature sources (e.g., OpenGrey) will be searched from inception to March 2025, with no language restrictions, leveraging translation services as needed. The search strategy, developed with an experienced librarian, will combine MeSH terms and keywords such as “anal fistula,” “fistula-in-ano,” “delayed wound healing,” “risk factors,” and “surgery”. Reference lists of relevant studies and prior reviews will be hand-searched for additional citations. Key search terms and databases are summarized in Table 3.

**Table 3:** Search Strategy for PubMed.

Concept	MeSH Terms	Free-Text Keywords	Boolean Operators
Anal Fistula	“Anal Fistula”[MeSH]	anal fistula, fistula-in-ano, perianal fistula	OR
Surgery	“Surgical Procedures, Operative”[MeSH]	surgery, fistulotomy, seton, flap, operative	OR
Delayed Wound Healing	“Wound Healing”[MeSH]	delayed healing, wound healing, epithelialization, non-healing	OR
Risk Factors	“Risk Factors”[MeSH]	risk, predictor, factor, association	OR
Combined Strategy		(#1 AND #2 AND #3 AND #4)	AND
Filters		Humans, adults (≥18 years), no date limit	

**Abbreviations**: MeSH, Medical Subject Headings. Search executed from inception to March 2025, with no language restrictions. Adapted for Embase, Web of Science, and grey literature searches.

### Study selection

Search results will be exported to EndNote X9 (Clarivate Analytics, 2020) for deduplication, then screening. Two independent reviewers will screen titles and abstracts against eligibility criteria, categorizing studies as “included,” “excluded,” or “maybe.” Discrepancies will be resolved by a third reviewer. Full-text screening will follow the same process, with reasons for exclusion documented.

### Data collection process

A standardized data extraction form will be piloted on a sample of eligible studies by two reviewers. Data extraction will be performed independently, with accuracy cross-checked and disagreements mediated by a third reviewer. Authors of original studies will be contacted for clarification if data are unclear or missing.

Extracted items will include: (1) study details (authors, year, country), (2) design (cohort/case-control), (3) participant characteristics (age, sex, comorbidities), (4) sample size, (5) risk factors assessed, (6) definition and incidence of delayed healing, (7) follow-up duration, (8) statistical methods, and (9) effect estimates (e.g., odds ratios [OR], relative risks [RR]). Data from figures will be extracted using WebPlotDigitizer (V.4.4) if necessary.

### Risk-of-bias assessment

The Newcastle-Ottawa Scale (NOS) will be used to assess risk of bias in included studies, evaluating selection, comparability, and outcome/exposure domains [39]. Each study will be rated as “low,” “moderate,” or “high” risk based on a score of 0–9, with thresholds of ≥7 (low), 4–6 (moderate), and <4 (high). Two reviewers will independently assess bias, resolving disagreements through consensus or third-party adjudication.

### Certainty of evidence

The Grading of Recommendations Assessment, Development and Evaluation (GRADE) framework, adapted for prognostic studies, will evaluate evidence quality [40]. Factors enhancing quality (e.g., large effect sizes) and reducing quality (e.g., inconsistency, imprecision) will be considered, with evidence graded as “high,” “moderate,” “low,” or “very low” for each risk factor.

### Data synthesis and meta-analysis

Quantitative synthesis will be pursued if sufficient data are available; otherwise, a narrative synthesis with structured tabulation will be conducted. Findings will be grouped by risk factor domains (e.g., patient-related, procedure-related).

### Accuracy of study selection

Inter-rater agreement for study selection and bias assessment will be calculated using the Kappa statistic with 95% confidence intervals, analyzed in SPSS V.29.0 [0, 2023].

### Summary statistics

Dichotomous outcomes (e.g., delayed healing incidence) will be expressed as pooled OR or RR with 95% CIs. Continuous outcomes (e.g., time to healing) will be reported as mean differences or standardized mean differences where applicable.

### Data synthesis

Meta-analysis will pool results from studies with homogeneous risk factors and outcomes, extracting unadjusted estimates from multifactor models where possible. Narrative synthesis will address multifactor models if individual effects cannot be isolated. The ‘meta’ package in R V.4.3.3 (R Foundation, 2024) will be used for meta-analysis, employing a random-effects model due to expected clinical heterogeneity. Forest plots will visualize pooled effects, with subgroup analyses by risk factor type if feasible. Heterogeneity will be assessed using the Q-statistic (p < 0.1 indicating significance) and I² test, with thresholds of 0–30% (low), 30–50% (moderate), 50–70% (considerable), and 70–100% (substantial) [41]. Forest plots will aid visual inspection, and subgroup analyses will explore sources of heterogeneity. If I² > 50%, subgroup analyses will examine study design, follow-up duration, and fistula complexity to clarify heterogeneity sources. Publication bias will be assessed using funnel plots and Egger’s test (p < 0.1 indicating bias), with the trim-and-fill method applied if bias is detected [42].

### Confirmation of risk factors

A risk factor will be confirmed as predictive if it shows a statistically significant association (p < 0.05) in ≥75% of studies with consistent directionality, following established criteria [43].

### Patients and public involvement

Patients and the public were not directly involved in designing this protocol due to its focus on synthesizing existing literature rather than primary data collection. However, the research question was informed by clinical priorities identified through patient forums and discussions with anorectal surgeons, highlighting the burden of DWH. No patients participated in recruitment or conduct, as this is a secondary analysis. Findings will be disseminated to patient advocacy groups via plain-language summaries and presentations at clinical conferences, ensuring accessibility and relevance to those affected by anal fistula surgery outcomes.

### Ethics and dissemination

Ethical approval is not required for this study, as it utilizes previously published data without involving human participants directly. The study poses no ethical risks, focusing solely on aggregating and analyzing existing evidence. Results will be disseminated through submission to a peer-reviewed journal and presented at international colorectal surgery conferences. Data will be made publicly available via PROSPERO, adhering to open-access principles to inform clinical practice and future research on anal fistula management.

### Amendments

Any amendments to this protocol will be documented and updated on PROSPERO (CRD420251013602) with a detailed rationale for changes, ensuring transparency. Modifications, such as adjustments to eligibility criteria, search strategy, or analysis methods, will be tracked with version numbers and dates. Significant changes will be reported in the final manuscript, including their impact on outcomes, following PRISMA-P guidelines. Minor updates will be noted in supplementary files (S1 Table).

## Discussion

### Expected main findings

This study seeks to delineate and quantify risk factors for DWH following anal fistula surgery, a complication observed in 10–30% of cases. Drawing on current evidence, we anticipate that patient-related factors—such as diabetes mellitus, smoking, and obesity—alongside procedure-related factors, including fistula complexity and postoperative infection, will emerge as robust predictors. Specifically, complex fistulas (e.g., high transsphincteric or branching tracts) are expected to exhibit elevated DWH rates due to their anatomical challenges, a finding supported by Kang et al.’s prospective cohort study, which reported higher healing delays in such cases [24]. Postoperative infections, documented in up to 25% of patients, are also likely to be significant, aligning with recent evidence from Bessa et al. [28]. We hypothesize that meta-analysis will substantiate these associations through pooled effect estimates, potentially uncovering dose-response relationships (e.g., smoking pack-years) or interactions (e.g., diabetes with infection). However, heterogeneity in DWH definitions—typically ranging from 6 to 12 weeks post-surgery—may complicate synthesis. To address this, subgroup analyses by study design and fistula type are planned to elucidate these effects. Ultimately, these findings will deliver a comprehensive risk profile, filling a critical gap in evidence synthesis and providing a foundation for targeted interventions in anal fistula management.

### Potential mechanisms

Elucidating the mechanisms driving DWH is pivotal to interpreting our anticipated findings. Diabetes impairs wound healing through microvascular damage and reduced angiogenesis, as evidenced by Dronge et al.’s study linking poor glycemic control to postoperative complications [44]. Studies further showed that hypoalbuminemia, often linked to hyperglycemia, disrupts wound repair, thereby delaying epithelialization [33,45]. Smoking, a well-established risk factor, compromises tissue oxygenation and fibroblast activity, with Sørensen’s systematic review highlighting nicotine’s vasoconstrictive effects [20]. Obesity contributes via chronic low-grade inflammation and adipokine dysregulation, as studies reported elevated interleukin-6 levels in obese surgical patients [46,47]. Complex fistulas are likely to prolong healing due to extensive tissue trauma and persistent microbial exposure [30]. Postoperative infections exacerbate delays by triggering inflammatory cascades that hinder granulation tissue formation [27]. Nutritional deficits, particularly hypoalbuminemia (<30 g/L), impair repair by limiting protein availability for tissue regeneration [33]. Collectively, these mechanisms point to a multifactorial etiology where systemic conditions amplify local surgical challenges. This study will assess the consistency of these pathways across studies, potentially identifying novel mediators—such as microbiome alterations [27].

### Comparison with previous similar studies

This protocol addresses a notable gap in the literature, as no prior systematic review has comprehensively synthesized risk factors for DWH after anal fistula surgery. Previous studies offer partial insights but lack the breadth of our approach. A meta-analysis by Mei et al. focused on recurrence rather than healing delays, reporting a pooled recurrence rate of 15–20% but overlooking patient-specific predictors [9]. Similarly, the network meta-analysis by An et al. evaluated 13 surgical techniques for complex anal fistulas, identifying fistulotomy as superior for healing speed yet omitting comorbidities like diabetes or smoking [25]. In contrast, our study integrates both patient-related (e.g., smoking, obesity) and procedure-related (e.g., fistula complexity, infection) factors, drawing on the most updated evidence from cohort studies. Additionally, unlike narrative reviews with pathogenesis overview [30], our use of meta-analytic methods and the GRADE framework will provide quantitative rigor and evidence quality assessment. This comprehensive synthesis, encompassing modifiable and non-modifiable risk factors, positions our study to advance beyond the fragmented evidence of prior work, offering a holistic foundation for clinical decision-making and future research in anal fistula surgery.

## Strengths and limitations

This protocol boasts several methodological strengths. Firstly, its exhaustive search strategy—spanning PubMed, Embase, Cochrane Library, Web of Science, and grey literature with no language barriers—ensures comprehensive coverage and minimizes selection bias. Secondly, employing the NOS for risk-of-bias assessment and the GRADE framework for evidence certainty adheres to gold-standard systematic review practices, bolstering reliability. Thirdly, by encompassing both patient- and procedure-related risk factors, this study extends beyond the scope of previous reviews, capturing modifiable predictors such as smoking and nutrition. The planned random-effects meta-analysis and subgroup analyses will effectively manage anticipated heterogeneity, delivering robust pooled estimates. Nevertheless, limitations warrant consideration. Variability in DWH definitions (e.g., > 6 weeks vs. > 12 weeks) may introduce inconsistency. Dependence on observational studies risks residual confounding despite NOS adjustments, and publication bias could skew results toward positive associations, though Egger’s test will mitigate this risk. Excluding Crohn’s disease-specific fistulas restricts generalizability to inflammatory contexts, a deliberate choice to focus on cryptoglandular fistulas. Furthermore, incomplete data from older studies or inaccessible grey literature may limit precision, though efforts to contact authors will address this.

### Clinical value and future directions

The clinical significance of this study lies in its potential to refine risk stratification and enhance perioperative care. Identifying predictors such as diabetes and smoking could drive preoperative optimization strategies, including glycemic control and smoking cessation programs, to reduce DWH incidence. Procedure-related factors, such as fistula complexity and infection, may inform tailored surgical approaches, such as enhanced antibiotic prophylaxis or minimally invasive techniques like seton placement. Nutritional interventions targeting hypoalbuminemia, shown to double complication risk, could become standard practice, offering a cost-effective means to improve outcomes. Economically, mitigating DWH could substantially lower healthcare costs, estimated at €5,561 per patient in Sweden, with delays doubling this burden [34]. Looking ahead, prospective cohort studies standardizing DWH definitions are needed to resolve current heterogeneity. Exploration of understudied factors, such as the cutaneous microbiome’s role, as proposed by Johnson et al. [27], or genetic markers, could refine risk models. Clinical trials targeting modifiable predictors—for instance, preoperative smoking cessation—could quantify their impact, while predictive models integrating multiple risk factors may pave the way for precision medicine in anal fistula care [11].

## Conclusions

This protocol outlines a systematic review and meta-analysis to synthesize risk factors for DWH following anal fistula surgery, addressing a pivotal evidence gap. We expect to confirm predictors offering a robust basis for risk reduction strategies that could decrease morbidity and healthcare costs. By directing future research toward standardized DWH definitions and novel predictors like microbiome influences, this study will catalyze advancements in the field.

## Supporting information

S1 Table PRISMA-P checklist.
(DOC)

## Abbreviations

ASCRS: American Society of Colon & Rectal Surgeons
BMI: Body Mass Index
CCF-IS: Cleveland Clinic Florida Incontinence Score
CRP: C-Reactive Protein
DWH: Delayed Wound Healing
EDC: Electronic Data Capture System
EMRI: Enhanced Magnetic Resonance Imaging
ESR: Erythrocyte Sedimentation Rate
GRADE: Grading of Recommendations Assessment, Development and Evaluation
HbA1c: Glycated Hemoglobin
HHAF: High Horseshoe-Shaped Anal Fistula
IBD: Inflammatory Bowel Disease
ITD: Incision and Thread-Drawing Therapy
LaC: Laser Closure
MITD-LaC: Multi-Incision and Tube-Dragging Therapy Combined with Laser Closure
MRI: Magnetic Resonance Imaging
NOS: Newcastle-Ottawa Scale
OR: Odds Ratio
PASS: Power Analysis and Sample Size Program
PRISMA-P: Preferred Reporting Items for Systematic Review and Meta-Analysis Protocols
QoLAF-QS: Quality of Life in Patients with Anal Fistula Questionnaire Score
RR: Relative Risk
SAS: Statistical Analysis System
SPSS: Statistical Package for the Social Sciences
TIVA: Total Intravenous Anesthesia
TRUS: Transrectal Ultrasonography
VAAFT: Video-Assisted Anal Fistula Treatment
VAS: Visual Analogue Scale
